# The effect of neoadjuvant chemotherapy among patients undergoing radical cystectomy for variant histology bladder cancer: A systematic review

**DOI:** 10.1080/2090598X.2021.1994230

**Published:** 2021-11-07

**Authors:** Mario Alvarez-Maestro, Francesco Chierigo, Guglielmo Mantica, J. M. Quesada-Olarte, D. M. Carrion, Juan Gomez-Rivas, Alvaro Pinto-Marin, Alfredo Aguilera Bazan, Luis Martinez-Piñeiro

**Affiliations:** a Department of Urology, Hospital Universitario La Paz, Madrid, Spain; b Department of Urology, Policlinico San Martino Hospital, University of Genova, Genoa, Italy

**Keywords:** Bladder cancer, neoadjuvant chemotherapy, systematic review, radical cystectomy, variant histology urothelial cancer

## Abstract

**Objective:**

To systematically review the evidence about the effect of neoadjuvant chemotherapy (NAC) for muscle-invasive bladder cancer (MIBC) with pure urothelial carcinoma (pUC) in radical cystectomy (RC) candidates affected by variant histology (VH) bladder cancer.

**Methods:**

A review of the current literature was conducted through the Medline and National Center for Biotechnology Information (NCBI) PubMed, Scopus databases in May 2020. The updated Preferred Reporting Items for Systematic Reviews and Meta-Analyses guidelines were followed for this systematic review. Keywords used were ‘bladder cancer’, ‘bladder carcinoma’, ‘bladder tumour’ and ‘bladder cancer variants’ and ‘neoadjuvant chemotherapy’. Only original articles in English published after 2000 and reporting oncological outcomes a series of more than five patients with VH were included. We excluded series in which the oncological outcomes of patients with pUC and VH were undistinguishable.

**Results:**

The literature search identified 2231 articles. A total of 51 full-text articles were assessed for eligibility, with 17 eventually considered for systematic review, for a cohort of 450,367 patients, of which 5010 underwent NAC + RC. The median age at initial diagnosis ranged from 61 to 71 years. Most patients received cisplatin-gemcitabine, methotrexate-vinblastine-adriamycin-cisplatin, or carboplatin-based chemotherapy. Only one study reported results of neoadjuvant immunotherapy. The median follow-up ranged from 1 to 120 months. The results showed that squamous cell carcinoma (SCC) is less sensitive to NAC than pUC and that SCC predicts poorer prognosis. NAC was found to be a valid approach in treating small cell carcinoma and may have potential benefit in micropapillary carcinoma.

**Conclusions:**

NAC showed the best oncological outcomes in small cell variants and micropapillary carcinoma, while NAC survival benefit for SCC and adenocarcinoma variants needs further studies. Drawing definite considerations on the efficacy of NAC in VH is complicated due to the heterogeneity of present literature. Present results need to be confirmed in randomised controlled trials.

## Introduction

Bladder cancer is the most common neoplasm of the urinary tract and worldwide the 11th most diagnosed cancer [1]. In most cases, bladder cancer is a pure urothelial carcinoma (pUC) but, in around 10–30% of patients, bladder cancer presents with a non-pure UC (npUC) or with pure variant histology (VH) without a UC component. Unlike pUC, the true prevalence, treatment options, and prognosis of these VHs are not well characterised. In an institutional study assessing the incidence of VH in patients treated with radical cystectomy (RC), 10.2% were found with squamous cell carcinoma (SCC), 8.3% with micropapillary carcinoma, 2.2% with glandular, 2.0% with sarcomatoid variant, 1.8% with small cell, 0.9% with lymphoepithelial, 3.2% with mixed variants, and 3.1% with other variants [2]. These findings are consistent with other two institutional studies, in which SCC, sarcomatoid, glandular and small cell were found to be the most common variants [3,4]. A population-based study conducted on Surveillance, Epidemiology and End Results (SEER) data, reported an annual incidence rate (calculated according to the total USA population) for SCC of 903 cases/year, 450 for adenocarcinoma and 447 for neuroendocrine tumours, while pUC was >50-times more common than any VH [5]. The impact of cisplatin-based neoadjuvant chemotherapy (NAC) before RC for high-grade muscle-invasive bladder cancer (MIBC) has been well established, with meta-analyses of prospective trials showing a 5–7% absolute overall survival (OS) [6–11]. No randomised controlled trials (RCTs) have focussed on the rarer and more aggressive VH subtypes of UC of the bladder. Prognosis is diverse for VH and there is a lack of evidence on the ideal treatment approach. Today, RC is still the ‘gold standard’ for both pUC MIBC and MIBC with VH [1]. In order to improve the low 5-year survival provided by RC; cisplatin-based NAC has been used over the last three decades. Nevertheless, the literature is scant regarding the impact of different rare variants on the response to NAC, while npUC histology has been found to have a different response to palliative systemic therapies [9]. Some of the few available studies regarding NAC found a survival benefit in patients with neuroendocrine tumours and in those with squamous and glandular differentiation [12]. On the contrary, the presence of bladder cancer VHs was associated with lower rates of response to NAC in other studies [13,14].

Considering this, the aim of the present systematic review was to provide the most recent and updated evidence about the effect of NAC in RC candidates with VH bladder cancer.

## Methods

### Literature search strategy

A review of the current literature was conducted through the Medline and National Center for Biotechnology Information (NCBI) PubMed, Scopus databases in May 2020. The updated Preferred Reporting Items for Systematic Reviews and Meta-Analyses (PRISMA) guidelines were followed for this systematic review [15].

Keywords used were: ‘bladder cancer’, ‘bladder carcinoma’, ‘bladder tumour’ and ‘bladder cancer variants’. We used the previous keywords as our primary search string, which combine established Medical Subject Headings (MeSH) terms for ‘neoadjuvant chemotherapy’ with the highly sensitive Cochrane search strategy. The reference lists of the retrieved reviews were also checked and cross-referenced [16].

The searches were performed independently by two researchers (F.C. and G.M.), and any disagreement resolved by a third independent researcher (M.A.M.). The initial screening was done on the base of titles and abstracts.

### Inclusion and exclusion criteria

All papers published after the year 2000, concerning studies conducted on humans for NAC for bladder cancer were considered for the review. Duplications were excluded using the dedicated tool on EndNote software. Only original articles (randomised controlled trials, cohort studies, case-control studies) for series of more than five patients were included. Other publications such as reviews, commentaries, editorials, and letters to the editor were excluded. The most recent publication was considered if several studies evaluated the same patient cohort. Only studies published in English were considered. Further, only studies reporting oncological outcomes of patients with rare HVs were included in the review. We also excluded series of both pUC and npUC patients in which the oncological outcomes of patients with pUC and of those with npUC could not be distinguished.

### Data extraction design

The overall risk of bias and Levels of Evidence (LoE) were assessed by the three reviewers using the Risk Of Bias In Non-randomised Studies – of Interventions (ROBINS-I) tool recommended by Cochrane and the Oxford Centre for Evidence-Based Medicine (OCEBM) criteria [17,18]. Variables that were recorded, when possible, included: variables related to the publication (year, country, design of the study); demographics (sample size, age, gender), histology at transurethral resection of bladder tumour (TURBT) or RC, TNM stage, NAC regimens given, type of loco-regional therapy, follow-up, oncological outcomes, and toxicity data with the complications/toxicity classification used.

### Statistical analysis

Data were entered into a Microsoft Excel (version 14.0) database and then transferred to Sofastat TM 1.4.6 for Windows. Descriptive statistics were calculated for all demographic, treatment, clinical and follow-up variables, and reported as median (interquartile range [IQR]) or as a proportion with percentage.

## Results

**Figure 1.**
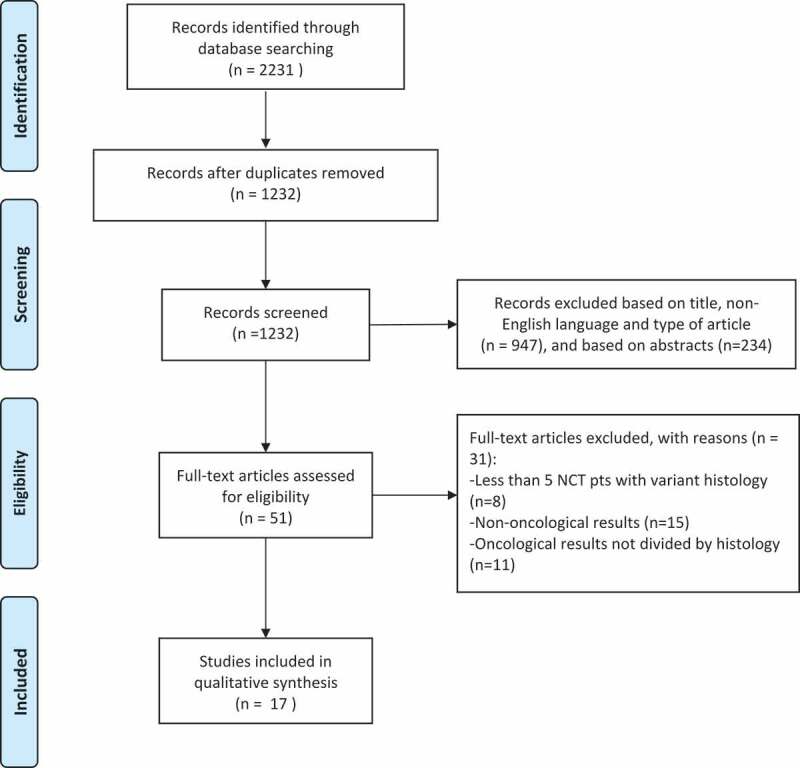
Flow diagram.

The PRISMA flow chart of the systematic review is presented in Figure 1. We identified 2231 articles from the Scopus and PubMed database searches. EndNote removed 999 duplications, 947 articles were discarded according to title, non-English language, and type of article (case reports, letters, editorials, reviews, etc.), and further 234 after reading the abstract. Overall, 51 full-text articles were assessed for eligibility: eight articles were excluded because they considered less than five patients with VH undergoing NAC, 15 because the articles did not report oncological results, and 11 because oncological results were not divided by histological type.

A summary of the variables is shown in Table 1. Of the 17 articles included in the present review, 13 were retrospective studies and three were clinical trials (Table 1) [13,14,19–33]. The median age at initial diagnosis ranged from 61 to 71 years and males greatly outnumbered females. The cohort of patients we considered comprised 450,367 patients, of which 5010 underwent NAC and RC. Due to the vast heterogeneity in the studies, it was quite difficult to understand how many patients with npUC underwent NAC + RC. The clinical stage of most of the population studied was cT2N0. Most patients received cisplatin-gemcitabine (CG), methotrexate-vinblastine-adriamycin-cisplatin (MVAC) or carboplatin-based chemotherapy, with only one reporting results of neoadjuvant immunotherapy. Unfortunately, five of 17 articles did not report the NAC regimens undergone and only one reported NAC-related toxicities. The median follow-up ranged from 1 to 120 months, averaging at ~40 months. Oncological results were also reported in quite different ways, mainly: downstaging after NAC, recurrence-free survival (RFS), cancer-specific survival (CSS), OS, recurrence rate, and mortality rate (MR) (Table 2) [13,14,19–33].

**Table 1.** Summary of study design and pre-therapy patients’ characteristics of the studies considered for review

Authors	Study design	Sample size	Age, years	Gender	Histology	TNM Stage	Notes
Bandini et al. [27]	R	950 (242 NAC)	68	NA	§206 pUC 36 npUC	cT stage cT2 = 59.9% cT3–4 = 37.6% Unknown = 2.5%	§Histology at TURBT
						pT stage pT0 = 32.6% pTa/pTis = 8.3% pT1 = 6.6% pT2 = 17.4% pT3 = 26.9% pT4 = 8.3%	
						pN Stage pNx = 1.2% pN0 = 81.4% pN1 = 8.3% pN2 = 8.7% pN3 = 0.4%	
Bandini et al. [29]	R	285 (450 NAC)	68	2331 Male 527 Female	**UC = 78% SCC = 9,9% MPUC = 3% ADC = 2,3% Small cell = 1,9% Sarcoma = 1,6% Other = 3,3%***	**cT stage <cT2 = 17.7% cT2 = 57.1% cT3-4 = 12.5% Unknown = 12.7%	*Histology at RC; **% of pts undergoing NAC + RC is not available; ***when considering npUC, % include both pure and combined VH
						cN stage cN0 = 43.6% cN1 = 5.4% cN2 = 3.6% cN3 = 0.4% cNx = 16.2% Unknown = 30.9%	
						pTN stage <pT2N0M0 = 14% pT0N0M0: 3.8% pT2N0M0 = 12.4% pT3-T4N0M0 = 27.5% pTanyN+M0 = 37.3% pTanyNx = 4.9%	
**Brimo et al**. [28]	**R**	**165**	**65**	**119 Male** **46 Female**	**UC = 76%** **npUC = 24%**	pT stage pT0 = 16% pTis = 14% pTa = 2% pT1 = 9% pT2 = 17% pT3 = 26% pT4 = 16%;	
						pN stage N0 = 68% N1 = 13% N2 = 15% N3 = 4%	
**Dotson et al**. [19]	**R**	**671** **(48 NAC)**	**61**	**NA**	**SCC**	cT stage cT2 = 75% cT3 = 25%	
						pT stage: <pT2 = 10.4% pT2 = 16.7% pT3 = 50% pT4 = 16.7% Unknown = 6.3% pN+ = 18.7%	
**Kamat et al**. [23]	**R**	**100** **(23 NAC)**	**66**	**21 Male** **3 Female**	**MPUC**	cT stage Tis = 4.4% Ta = 0% T1 = 39.1% T2 = 30.4% T3 = 13.0% T4a = 13%	
						**pT stage** **pT0 = 39.1%** **pTis = 17.4%** **pT1 = 4.4%** **pT2 = 4.4%** **pT3 = 17.4%** **pT4 = 4.4%** **N+ = 13%**	
**Lin et al**. [30]	**R**	**195** **(37 NAC)**	**66**	*7 Male **5 Female**	**pUC = 161** **npUC = 34**	*cT stage **cT2 = 58%** **cT3 = 25%** **cT4 = 17%**	*N° referring to pts with npUC undergoing NC
**Lynch et al**. [26]	**R**	**172** **(48 NAC)**	**67**	**41 Male** **7 Female**	**SC only = 22** **>50% SC = 18** **<50% SC = 8**	**cT stage** **≤ cT1N0 = 1** **cT2N0 = 30** **cT3-4aN0 = 17**	
**Matulay et al**. [20]	**R**	**260** **(75 NAC)**		**NA**	*UC = 3896 **SCC = 75**	**cT2-4N0M0**	* N° referring to NAC + RC patients
**Meeks et al**. [24]	**R**	**44** **(29 NAC)**	**71**	**26 Male** **18 Female**	**MPUC**	**NA**	
**Minato et al**. [22]	**R**	**38**	**66**	**29 Male** **9 Female**	**UC = 29** **SCC = 9**	**UC** **T3 = 93.6%** **T4 = 3.4%**	
						**SCC** **T3 = 88.9%** **T4 = 11.1%**	
**Necchi et al**. [33]	**P-II CT**	**114** **(111 NAC)**	**66**	**99 Male** **15 Female**	**pUC = 80**	**cT stage** **T2 = 54%** **T3 = 44%** **T4 = 3.6%**	
					**npUC = 34** **33% SCC** **17% nested** **12% MPUC** **9% LEL**, **6% sarcomatoid** **6% ADC+SCC** **3% ADC** **3% small cell** **3% plasmacytoid** **3% spindle cell** **3% clear cell** **3% SCC+SC**		
**Pokuri et al**. [14]	**R**	**50**	**67,5**	**41 Male** **9 Female**	**pUC = 52%** **npUC = 48%**	**Clinical stage** **cT2N0 = 62%** **cT3N0 = 22%** **cT4N0 = 16%**	
**Scosyrev et al**. [13]	**CT**	**307** **(124 NAC)**	**VH** **NAC + RC = 60**	**VH – NAC + RC** **69% Male** **31% Female**	**pUC = 236** **SCC = 37** **ADC = 20** **SCC+ADC = 2** **Other = 2**	**VH** **cT3–cT4a = 59%**	*patient % relative to those who received NAC + RT
			**VH** **RC = 65**	**VH – RC 85% Male** **15% Female**			
			**pUC** **NAC + RC = 63**	**pUC – NAC + RC** **86% Male** **14% Female**		**pUC** cT3–cT4a = 59%*	
			**pUC** **RC = 62**	**pUC + RC** **79% Male** **21% Female**			
**Siefker-Radtke et al**. [31]	**P-II CT**	**65**	**62,5**	**50 Male** **15 Female**	**pUC = 57%** **VH = 43%** (MPUC 46% of VH)*	**cT stage of bladder/urethra tumor (n = 60)** **cT2 = 37** **cT3b = 18** **cT4a = 5**	*VH ≤50% of the specimen
						**Tumor in the renal pelvis or ureter = 5**	
**Stensland et al**. [21]	**R**	**828** **(53 NAC)**	**63,5**	**29 Male** **16 Female**	**SCC**	**cT stage** **T2 = 79.2%** **T3 = 20.8%**	
**Sui et al**. [25]	**R**	**439188** **(3083 NAC)**	**pUC = 71,1**	**pUC** **Male 75.4%** **Female 24.6%**	*UC = 3052 **MPUC = 31**	**MPUC** **cT stage** **Tx 17.4%** **T0 = 0%** **Tis = 5.2%** **T1 = 29.8%** **T2 = 35.4%** **T3 = 7.1%** **T4 = 5.1%** **cN stage** **N0 = 70%** **N+ = 9.1%** **Nx = 20.9%** **cM stage** **M0 = 95.3%** **M1 = 4.7%**	* N° referring to pts undergoing NAC + RC
			**MPUC = 69.9**	**MPUC** **Male 78.3%** **Female 21.7%**		**UC** **cT stage** **Tx 16.5%** **T0 0.3%** **Tis 47.2%** **T2 11.5%** **T3 1.6%** **T4 1.8%** **cN stage** **N0 77.4%** **N + 1.8%** **Nx 20.8%** **cM stage** **M0 98.1%** **M1 1.9%**	
**Vetterlein et al**. [32]	**R**	**2018** **(369 NAC)**	**66,7**	**Male 62.4%** **Female 37.4%**	**MPUC = 7.6%** **sarcomatoid = 15.1%** **SCC = 40.1%** **ADC = 17.7%** **NE = 13.3%** **other = 6.1%**	**cT2N0 = 65.1%** **≥cT2 and/or cN1 = 34.9%**	

ADC: adenocarcinoma; NE: neuroendocrine; P-II CT: phase II clinical trial; R: retrospective; SC: small-cell.

**Table 2.** Summary of NAC regimens, locoregional therapy, length of follow-up, oncological outcomes and toxicity of the studies considered for review

Authors	NAC regimens	Loco-regional therapy	Follow-up, months	Oncological outcomes	Notes
Bandini et al. [27]	*Carboplatin: 20 (8.3%) Cisplatin 203 (83.9%) Unknown: 19 (7.9%) Median NAC cycles = 3	RC	26	1-year RFS = 76,9%	*% relative to patients undergoing NAC in the study. At univariate Cox regression, npUC did not predict recurrence after RC.
Bandini et al. [29]	**Carboplatin = 1.6% CMV/MVEC = 0.7% GC = 6.9% MVAC = 3.4% no NAC = 77.2% Other = 3.3% Unknown = 7% RT to the primary = 5%	RC	29	§CSS UC = 116 months SCC = 33 months MPUC = 28 months ADC = 107 months SC = 46 months Sarcoma = 12 months***	**% of patients undergoing NAC + RC is not available; ***when considering npUC, % include both pure and combined VH; §median CSS of patients receiving NAC + RC
Brimo et al [28]	GC = 68% MVAC = 32%	RC	3–120	disease progression in 45% at mean FU of 19.6 months cancer related deaths in 30% at a mean FU of 21.6 months	At multivariate analysis VH resulted a predictor of recurrence but not of survival
Dotson et al [19]	NA	RC	31.9	2-year OS (RC vs NAC + RC) = 54.8% vs 45.7%	
Kamat et al [23]	NA	RC	1–182	Pathological downstaging 63% Pathological upstaging 21% 5-year OS = 63%	
Lin et al [30]	*MVAC = 42% GC = 25% Gemcitabine+carboplatin = 33%	RC	18	Downstaging = 16.7% In the group of npUC, NAC did not confer a significant survival benefit.	*N° referring to patients with npUC undergoing NC
Lynch et al [26]	ifosfamide+doxorubicine+etoposide+cisplatin = 54% etoposide+cisplatin = 15% MVAC = 10% paclitaxel+methotrexate+cisplatin = 6% cisplatin+gemcitabine+ifosfamide = 4% etoposide+doxorubicin+cisplatin = 4% ifosfamide+doxorubicine = 2% gemcitabine+cyclophosphamide = 2% gemcitabine+doxorubicin+paclitaxel = 2%	47 RC 1 M+	NA	Median OS = 159.5 months 5-year CSS = 79% Downstaging = 62%	
Matulay et al [20]	NA	RC	NA	Median OS SCC – RC alone = 25.4 months SCC – NAC + RC = 34.0 months (*P* = 0.34)	
Meeks et al [24]	GC = 21 GC+sunitinib = 2 gemcitabine+carboplatin = 2 paclitaxel+GC = 3 MVAC = 1	RC = 93% PC = 7%	28	NAC+RC vs RC alone 2-year recurrence rate = 33% vs 62% (*P* > 0.05) 2-year CSM = 66% vs 57% (*P* > 0.05) 2-year OM = 71% vs 84% (*P* > 0.05) Downstaging to pT0 = 45% vs 13% pT0 had better OS, CSS and RFS.	
Minato et al [22]	UC MVAC = 62.1% GC = 37.9%	RC	UC 48	Recurrence (SCC vs UC) = 88.9% vs 48.3% Deaths (SCC vs UC) = 77.8% vs 48.3%	
	SCC MVAC = 22.2% GC = 77.8%		SCC 16		
Necchi et al [33]	Pembrolizumab	RC	13.2	*pT0 rate = 37% vs 16% vs 53% vs 39% pT ≤1 rate = 55% vs 42% vs 67% vs 56% In SCC, pT ≤1 rate = 86% In LEL pT0 rate = 67%.	(Overall – predominant VH – non-predominant VH – pUC)
Pokuri et al [14]	Ciplatin based = 96% non-cisplatin based = 4%	RC	NA	Histological type only predictor of pT0 response to NAC OR 0.09, 95% CI 0.021–0.380), *P* = 0.001	
Scosyrev et al [13]	MVAC	RC	NA	5-year OS (pUC vs npUC) cT2 RC only = 61% vs 54% cT2 NAC + RC = 64% vs 73% cT3-4a RC only = 42% vs 34% cT3-4a NAC + RC = 46% vs 58%	
Siefker-Radtke et al [31]	3X Ifosfamide, Doxorubicin, Gemcitabine + 4X Cisplatin, Gemcitabine, Ifosfamide.	RC	85.3	5-year CSS npUC = 50% pUC = 83% (*P* > 0.05) For MPUC, 5-year CSS = 54% and OS = 54%	
Stensland et al [21]	NA	RC	11.2	RC alone and NAC + RC did not differ.	
Sui et al [25]	NA	RC	NA	No survival difference between NAC and RC alone observed in patients with ≥cT2 MPUC	
Vetterlein et al [32]	NA	RC	50.9	Median OS (months, NAC+RC vs RC only) MPUC = 51.7 vs 29.0 Sarcomatoid = 27.1 vs 15.0 SCC = 26.2 vs 25.4 ADC = 37.2 vs 32.0 NE 34.7 vs 17.3* Other = NA vs 71.9 *statistically significant OS benefit for NAC only in patients with NE tumours.	

ADC: adenocarcinoma; NE: neuroendocrine; SC: small-cell.

### Squamous cell carcinoma (SCC)

Three studies focussed on SCC. Using data from the National Cancer Database (NCDB), Dotson et al. [19] evaluated the management and survival of patients with invasive SCC treated with or without NAC. Although more patients were down-staged to non-invasive disease (pT < 2) in the NAC group, the 2-year OS was not statistically significantly different, being 54.8% for RC alone and 45.7% for NAC + RC.

The results are consistent with those by Matulay et al. [20], who, still using the NCBD, showed that the median OS for patients with SCC who received RC alone was 25.4 months compared to 34.0 months with NAC, although not statistically significant.

The NCDB was queried once again by Stensland et al. [21] for cases of localised, muscle-invasive pure squamous cell bladder cancer, classified as clinical stage T2/3N0M0. In this study, the unweighted median survival was 46 months for RC alone, and 21 months for NAC + RC. However, in the weighted multivariate Cox model, compared to RC alone, NAC did not significantly differ with regard to OS.

One major limitation of these three studies is that the NCDB lacks specific data on chemotherapy: the type, duration, and dose of chemotherapy cannot be analysed, so NAC is probably a heterogeneous group comprising multiple types of chemotherapeutic regimens.

A study from Japan also tried to investigate the efficacy of cisplatin-based chemotherapy (MVAC or CG) and prognosis of patients with UC with or without squamous differentiation of the bladder. In this study, none of the patients with SCC achieved pathological complete response (pCR) to NAC and the proportion of down-staging was lower in patients with SCC than in ones with pUC [22]. The median follow-up was 16 and 48 months in patients with SCC and pUC, respectively. After RC, recurrence developed in 88.9% and 48.3% of the patients with SCC and pUC, respectively; and 77.8% and 48.3% died in the SCC and pUC groups, respectively.

From these results, it appears that SCC is less sensitive to NAC than pUC, and that SCC predicts poorer prognosis. However, there is a need for larger, prospective investigations, with design confronting different NAC regiments in patients with SCC.

### Micropapillary carcinoma

We identified three studies focussing on micropapillary carcinoma. Kamat et al. [23] retrospectively reviewed data of 100 patients with micropapillary carcinoma, 23 of them undergoing NAC + RC. They observed a 5‒year OS rate of 63% in patients treated with NAC + RC, which was not significantly different from what was observed for the patients treated with upfront RC, who had a 5-year OS rate of 71%. Interestingly, patients who had non-MIBC (NMIBC), delaying surgery for neoadjuvant therapy demonstrated a trend toward a decreased median survival and 5-year survival rate compared to upfront surgery.

Another study reported that the rates of recurrence (33% vs 62%), cancer‐specific mortality (66% vs 57%) and overall mortality (71% vs 84%) were not different at 2 years between patients who did or did not receive NAC. However, down‐staging to pT0 occurred in 45% of patients receiving NAC, compared with 13% of those who underwent upfront RC (*P* = 0.049). Despite micropapillary histology, those with pT0 had improved outcomes after RC, with a lower rate of recurrence, lower bladder cancer-specific mortality and longer time to death. Therefore, the authors [24] concluded that patients with the micropapillary variant of UC should not be excluded from consideration for NAC.

Sui et al. [25] analysed data of from the NCBD, in which 94 patients with ≥cT2 disease were identified. In this study, there was not a statistically significant difference in median OS between patients who received NAC and those who did not.

### Small cell carcinoma

Lynch et al. [26] identified 125 patients with small cell UC with a clinical stage ≤cT4aN0M0. Of these, 95 were surgical candidates: 48 received NAC and 47 underwent upfront RC. Neoadjuvant treatment was associated with improved OS and CSS compared with initial RC (median OS: 159.5 vs 18.3 months, *P* < 0.001; 5-year CSS: 79% vs 20%, *P* < 0.001). NAC resulted in pathological downstaging to ≤pT1N0 in 62% of tumours compared with only 9% treated with initial RC. Although limited by a small sample size with a retrospective analysis, and this being the only article dealing solely with small cell carcinoma, these data suggest NAC as a valid approach in treating small cell carcinoma of the bladder.

### Articles studying more than one npUC histology

The other nine studies we included in the present review considered npUC without discriminating for histological type or considered many histological variants.

In 2019, Bandini et al. [27], in a study aimed at modelling 1-year RFS after NAC + RC in patients with cT2–4N0M0 bladder cancer, showed that npUC was not a predictor of recurrence after RC at univariable Cox regression model analysis. On the contrary, a multicentric study which also investigated prognostic pathological factors in RC after NAC showed that VH was a predictor of recurrence, but not of cancer-related death [28].

In 2020, a multi-institutional study aimed to examine the effect of NAC on bladder cancer with different histological variants [29]. Of the 450 NAC-treated patients, only patients with SCC had had worse CSS (median CSS, 33 vs 116 months; *P* < 0.001) and higher mortality rates (hazard ratio [HR] 2.1; *P* = 0.03) compared with those with pUC. After adjusting for NAC, only SCC showed a lower rate of clinical-to-pathological downstaging (odds ratio [OR] 0.4; *P* = 0.03) compared with UC.

In 2012, Lin et al. [30] examined the effects of NAC in the treatment of MIBC in patients with npUC vs those with pUC. In their study, the rate of downstaging to pT0 was higher in NAC-treated patients with both npUC (*P* = 0.048) and pUC (*P* < 0.001), as compared to those in each group who did not receive NAC. However, NAC was not a significant predictor of OS for patients with npUC in a Cox multivariate model (*P* = 0.54) and, among all patients treated with NAC, mixed histology was found to be a predictor of poorer survival.

In addition, Pokuri et al. [14] found that the odds of a pT0 response for pUC were approximately 11-times greater relative to cancers with VH features or mixed tumours (OR 0.09, 95% CI 0.021–0.380; *P* = 0.001), including squamous, glandular differentiation, small cell, micropapillary, sarcomatoid, nested component, lymphoepithelioma-like (LEL), and plasmacytoid variants.

A secondary analysis of the Southwest Oncology Group (SWOG)-directed intergroup randomised trial S8710 of neoadjuvant MVAC followed by RC vs RC alone for treatment of locally advanced UC of the bladder gave evidence of a survival benefit from chemotherapy in patients with mixed tumours (HR 0.46, 95% CI 0.25–0.87; *P* = 0.02). Moreover, there was marginal evidence that the survival benefit of NAC in patients with mixed tumours was greater than it was for patients with pUC. These analyses also showed that the estimated improvement in 5-year survival associated with MVAC was much greater in magnitude among patients with npUC than among patients with pUC [13].

A phase II clinical trial of sequential NAC with ifosfamide, doxorubicin, and gemcitabine, followed by cisplatin, gemcitabine, and ifosfamide showed that the presence of VH was associated with an inferior 5-year CSS of 50% as compared to 83% for pUC (log-rank *P* = 0.02). In this series, the presence of micropapillary histology was associated with a 5-year OS and CSS of 54%. A pUC histology was also a significant factor to disease-specific survival (relative risk 0.35 for UC vs mixed, *P* = 0.03). This clinical trial was the only study included in our review to report NAC-related toxicities. There was only one death due pneumonia during neutropenia. In all, 6% of patients had Grade 4 toxicities (myocardial infarction, platelet transfusion, and vomiting), while the most frequent Grade 3 toxicities were infection (38%), febrile neutropenia (22%), and mucositis 18%, and platelet transfusion (12%) [31].

In 2017, Vetterlein et al. [32] assessed the effect of NAC on OS after RC in patients with HVs, finding an OS benefit for NAC only in patients with neuroendocrine tumours (HR 0.49, 95% CI 0.33–0.74; *P* = 0.001). For other HVs, even if NAC decreased the frequency of non-organ confined disease at the time of RC, this did not translate into a statistically significant OS benefit.

Taken together, these results show that classic NAC regimens have only a modest role in MIBC with VH, often not providing a survival benefit. Fortunately, preliminary findings on the activity of neoadjuvant pembrolizumab in patients with MIBC and predominant VH move to the direction of broadening the inclusion criteria of neoadjuvant immunotherapy trials also to this kind of patient. Indeed, in a very recent article, Necchi et al. [33] showed that even if an overall substantially lower activity of pembrolizumab was found in patients with predominant VH, in patients with predominant VH, a substantially lower activity of pembrolizumab was found, as the pT0 rate was 16% (95% CI 3.4–40) and the pT ≤1 rate was 42% (95% CI 21–67). However, there was significant heterogeneity in this group: in fact, six of seven patients with SCC achieved a pT ≤1 response, and two of three patients with a LEL variant achieved a pT0 response (the remaining patient refused RC but obtained a clinical T0 response on re-TURBT).

## Discussion

The standard of care for MIBC is NAC followed by RC and pelvic lymph node dissection. A pCR occurs in a wide range of cases (20–35%), and tumour downstaging to NMIBC is obtained in ~50% of cases. Unfortunately, several limitations affected the use of NAC in clinical practice, and patients who have residual MIBC after RC have a high likelihood of relapse and death from metastatic bladder cancer.

Cisplatin-based NAC followed by RC and pelvic lymph node dissection is the standard of care for cisplatin-eligible patients. There is no currently approved NAC for cisplatin-ineligible patients, so these patients should directly undergo RC. There is no role of carboplatin and gemcitabine combination. Dose-dense (dd) or accelerated MVAC was evaluated as NAC following a modest size randomised phase III trial demonstrating improved long-term survival compared to conventional MVAC in UC. Three or four cycles of dd-MVAC were tested in combination with granulocyte growth factor support in the neoadjuvant setting in two smaller non-randomised phase II trials with results similar to those observed in SWOG 8710 [34,35]. In a real-world experience study of dd-MVAC followed by RC, 345 patients were included with 85% having high-risk features (HRFs) including cT3–T4 disease, hydronephrosis, and VH. In all, 30% of the patients had pCR (pT0N0) and 49.3% patients with ≤pT1N0.

The role of NAC in VH bladder cancers has yet to be validated in RCTs. Several case series have reported experiences with NAC in the setting of VH. The impact of cisplatin-based NAC before RC for high-grade MIBC has been well established, with RCTs showing improved survival outcomes. Regimens of MVAC or CG have been shown to increase OS with an absolute benefit of 5%. Despite these studies showing a clear survival benefit, no RCTs have focussed on the rarer and more aggressive VH subtypes of UC of the bladder. These may include UCs with any component of VH subtype, or purely VH subtypes with no UC component. Prognosis is diverse for VH and there is a lack of evidence on the ideal treatment approach. Around 25% of MIBC cases have associated variant morphologies. While patients with a minor VH component are treated like conventional UC, there are no definitive data to guide the therapy of those with a major or pure variant component. Multiple studies have suggested that some HVs are associated with adverse pathological features and outcomes, particularly micropapillary, plasmacytoid, and small-cell histology [2,36]. However, other data suggest that only the pure variants predominantly micropapillary or small cell and not mixed variant histologies mostly with squamous, adenocarcinoma, sarcomatoid, and lymphoepithelioma components were associated with poor outcomes compared to pUC patients [37]. According to our present systematic review, there is evidence supporting NAC with cisplatin and etoposide for neuroendocrine/small-cell tumours [26], although controlled data similar to small-cell lung cancer does not exist. In UC with squamous and glandular differentiation, the data on NAC are controversial: some studies have demonstrated an advantage with the downstaging of disease while others have shown a poor response. Interestingly, patients with predominant SCC VH achieved 86% pT ≤1 response rate and 67% of LEL variant patients achieved a pT0 response with neoadjuvant pembrolizumab, suggesting that these variants may be more sensitive to immunotherapy [33]. In micropapillary UC (MPUC), due to paucity of data, the recommendations include immediate RC or NAC followed by RC [22–24]. In plasmacytoid UC, the role of NAC is unclear due to small retrospective studies with differing chemotherapy regimens.

Although some studies have shown it to be chemo-sensitive, others have suggested that even after achieving pCR following NAC, survival, and prognosis remains poor [38]. Similarly, in sarcomatoid UC, the lack of benefit of NAC has been shown in multiple studies [32]. Indeed, a survival benefit of adjuvant chemotherapy has also not been identified for patients who had UC with concomitant variant or pure VH [39].

The effect of chemotherapy on VH bladder cancer has also been assessed with trimodal bladder-sparing therapy comprising radiation, chemotherapy, and maximal TURBT. One study grouped all VHs together and found that the complete response rate after induction chemoradiation was 82% compared to 83% in pure UC [40]. There was a non-significant difference in the 5- and 10-year OS rates (52% and 42% for VH vs 61% and 42% for pUC). The unique nature of VH bladder cancers suggests a need to identify treatment plans tailored to specific VHs. Several studies have evaluated the role of NAC in this setting with varying results for each VH, indicating that other novel methods, such as genome sequencing, may provide further direction on the appropriate use of NAC. Studies have shown that p53-like bladder cancers are consistently resistant to NAC while cancers with mutations in fibroblast growth factor receptor 3 (FGFR3) may respond to targeted therapies, suggesting that the use of NAC may be aided by gene expression profiling [41].

Data suggest that precision medicine by selecting patients likely to benefit with cisplatin-based NAC may be possible with further validation. Today, distinct genomic alterations in DNA damage response pathways and transcriptomic molecular subtypes in MIBC have been linked with varied response to NAC, suggesting that precision medicine may be possible. Patients harbouring tumours without the sensitive molecular alterations (genomic or transcriptomic) may also exhibit pCR, suggesting that these assays cannot currently be used to deny cisplatin-based NAC to cisplatin-eligible patients off-trial [42]. However, while these platforms require optimal prospective validation to enable their routine use in the clinic, there are still several existing challenges due to tumour heterogeneity, clonal evolution with treatment, assay result turnaround time and costs, making many of these tests impractical to use in current routine clinical practice. Nevertheless, non-randomised trials are ongoing to select patients with sensitising genomic alterations and MIBC who attain clinical CR with cisplatin-based NAC for a bladder-sparing approach (NAC02710734, NAC03609216). Moreover, given the promising pCR with the combination of cisplatin-based chemotherapy and programmed cell death-protein 1/-ligand 1 (PD-1/-L1) inhibitors, multiple phase III trials are evaluating this strategy in cisplatin-eligible patients with MIBC [43].

## Conclusions

Several case series have reported experiences with NAC in the setting of VH. Outcomes varied significantly in the current literature. The best outcomes are associated with NAC for small-cell and micropapillary variants, while there is potential benefit with the use of NAC for squamous cell and adenocarcinoma variants. Molecular sub-classification and development of predictive biomarkers in MIBC will further help to identify optimal treatment strategies in these patients. The role of NAC in VH bladder cancers has yet to be validated in RCTs.

## References

[1] Witjes JA, Bruins HM, Cathomas R, et al. European association of urology guidelines on muscle-invasive and metastatic bladder cancer: summary of the 2020 guidelines. Eur Urol. 2021;82–104. 10.1016/j.eururo.2020.03.055 32360052

[2] Moschini M, Dell’Oglio P, Luciano R, et al. Incidence and effect of variant histology on oncological outcomes in patients with bladder cancer treated with radical cystectomy. Urol Oncol Semin Orig Investig. 2017;35:335–341. Available from: https://pubmed.ncbi.nlm.nih.gov/28087131/10.1016/j.urolonc.2016.12.006 28087131

[3] Soave A, Schmidt S, Dahlem R, et al. Does the extent of variant histology affect oncological outcomes in patients with urothelial carcinoma of the bladder treated with radical cystectomy? Urol Oncol Semin Orig Investig. 2015;33:21.e1–21.e9. 10.1016/j.urolonc.2014.10.013 25465301

[4] Xylinas E, Rink M, Robinson BD, et al. Impact of histological variants on oncological outcomes of patients with urothelial carcinoma of the bladder treated with radical cystectomy. Eur J Cancer. 2013;49:1889–1897. 23466126 10.1016/j.ejca.2013.02.001

[5] Deuker M, Martin T, Stolzenbach F, et al. Bladder cancer: a comparison between non-urothelial variant histology and urothelial carcinoma across all stages and treatment modalities. Clin Genitourin Cancer. 2020;19:60–68.e1. 32782133 10.1016/j.clgc.2020.07.011

[6] Winquist E, Kirchner TS, Segal R, et al. Neoadjuvant chemotherapy for transitional cell carcinoma of the bladder: a systematic review and meta-analysis. J Urol. 2004;171:561–569. 14713760 10.1097/01.ju.0000090967.08622.33

[7] Vale C. Neoadjuvant chemotherapy in invasive bladder cancer: a systematic review and meta-analysis. Lancet. 2003;361:1927–1934. 12801735 10.1016/s0140-6736(03)13580-5

[8] Vale CL. Neoadjuvant chemotherapy in invasive bladder cancer: update of a systematic review and meta-analysis of individual patient data advanced bladder cancer (ABC) meta-analysis collaboration. Eur Urol. 2005;48:202–205; discussion 205–206. 10.1016/j.eururo.2005.04.006 15939524

[9] Yin M, Joshi M, Meijer RP, et al. Neoadjuvant chemotherapy for muscle‐invasive bladder cancer: a systematic review and two‐step meta‐analysis. Oncologist. 2016;21:708–715. [cited 2021 Jan 17]. Available from: https://pubmed.ncbi.nlm.nih.gov/27053504/27053504 10.1634/theoncologist.2015-0440 PMC4912364

[10] Grossman HB, Natale RB, Tangen CM, et al. Neoadjuvant chemotherapy plus cystectomy compared with cystectomy alone for locally advanced bladder cancer. N Engl J Med. 2003. 10.1056/NEJMoa022148 12944571

[11] Griffiths G. International phase III trial assessing neoadjuvant cisplatin, methotrexate, and vinblastine chemotherapy for muscle-invasive bladder cancer: long-term results of the BA06 30894 trial. J Clin Oncol. 2011;29:2171–2177. 21502557 10.1200/JCO.2010.32.3139 PMC3107740

[12] Geynisman DM, Handorf E, Wong YN, et al. Advanced small cell carcinoma of the bladder: clinical characteristics, treatment patterns and outcomes in 960 patients and comparison with urothelial carcinoma. Cancer Med. 2016;5:192–199. 26679712 10.1002/cam4.577 PMC4735777

[13] Scosyrev E, Ely BW, Messing EM, et al. Do mixed histological features affect survival benefit from neoadjuvant platinum-based combination chemotherapy in patients with locally advanced bladder cancer? A secondary analysis of Southwest Oncology Group-Directed Intergroup Study (S8710). BJU Int. 2011;108:693–699. [cited 2021 Jan 18]. Available from: https://pubmed.ncbi.nlm.nih.gov/21105991/21105991 10.1111/j.1464-410X.2010.09900.x PMC3117124

[14] Pokuri VK, Syed JR, Yang Z, et al. Predictors of Complete Pathologic Response (pT0) to neoadjuvant chemotherapy in muscle-invasive bladder carcinoma. Clin Genitourin Cancer. 2016;14:e59–e65. [cited 2021 Jan 18]. Available from: https://jhu.pure.elsevier.com/en/publications/predictors-of-complete-pathologic-response-pt0-to-neoadjuvant-che 26508364 10.1016/j.clgc.2015.09.013

[15] Moher D, Liberati A, Tetzlaff J, et al. Preferred reporting items for systematic reviews and meta-analyses: the PRISMA statement. PLoS Med. 2009;6:e1000097. DOI:10.1371/journal.pmed.1000097 19621072 PMC2707599

[16] Higgins JP, Green S editors. Cochrane handbook for systematic reviews of interventions. Chichester (UK): John Wiley & Sons, Ltd; 2008. DOI:10.1002/9780470712184

[17] Sterne JA, Hernán MA, Reeves BC, et al. ROBINS-I: a tool for assessing risk of bias in non-randomised studies of interventions. BMJ. 2016;355. DOI:10.1136/bmj.i4919. PMC5062054 27733354

[18] OCEBM. Levels of evidence — centre for Evidence-Based Medicine (CEBM). University of Oxford. [cited 2021 Jan 18. [cited 2021 Jan 18]. Available from: https://www.cebm.ox.ac.uk/resources/levels-of-evidence/ocebm-levels-of-evidence.

[19] Dotson A, May A, Davaro F, et al. Squamous cell carcinoma of the bladder: poor response to neoadjuvant chemotherapy. Int J Clin Oncol. 2019;24:706–711. 30707342 10.1007/s10147-019-01409-x

[20] Matulay JT, Woldu SL, Lim A, et al. The impact of squamous histology on survival in patients with muscle-invasive bladder cancer. Urol Oncol Semin Orig Investig. 2019;37:353.e17–353.e24. [cited 2021 Jan 18]. Available from: https://pubmed.ncbi.nlm.nih.gov/30704959/10.1016/j.urolonc.2019.01.020 30704959

[21] Stensland KD, Zaid H, Broadwin M, et al. Comparative effectiveness of treatment strategies for squamous cell carcinoma of the bladder. Eur Urol Oncol. 2020;3:509–514. [cited 2021 Jan 18]. Available from: https://pubmed.ncbi.nlm.nih.gov/31411987/31411987 10.1016/j.euo.2018.11.003

[22] Minato A, Fujimoto N, Kubo T. Squamous differentiation predicts poor response to cisplatin-based chemotherapy and unfavorable prognosis in urothelial carcinoma of the urinary bladder. Clin Genitourin Cancer. 2017;15:e1063–e1067. 28803791 10.1016/j.clgc.2017.07.008

[23] Kamat AM, Dinney CPN, Gee JR, et al. Micropapillary bladder cancer: a review of the University of Texas M. D. Anderson Cancer Center experience with 100 consecutive patients. Cancer. 2007;110:62–67. [cited 2021 Jan 18]. Available from: https://pubmed.ncbi.nlm.nih.gov/17542024/17542024 10.1002/cncr.22756

[24] Meeks JJ, Taylor JM, Matsushita K, et al. Pathological response to neoadjuvant chemotherapy for muscle-invasive micropapillary bladder cancer. BJU Int. 2013:111. [cited 2021 Jan 18]. Available from: https://pubmed.ncbi.nlm.nih.gov/23384236/10.1111/j.1464-410X.2012.11751.x 23384236

[25] Sui W, Matulay JT, James MB, et al. Micropapillary bladder cancer: insights from the national cancer database. Bl Cancer. 2016;2:415–423. [cited 2021 Jan 18]. Available from: https://pubmed.ncbi.nlm.nih.gov/28035322/28035322 10.3233/BLC-160066 PMC5181670

[26] Lynch SP, Shen Y, Kamat A, et al. Neoadjuvant chemotherapy in small cell urothelial cancer improves pathologic downstaging and long-term outcomes: results from a retrospective study at the md anderson cancer center. Eur Urol. 2013;64:307–313. [cited 2021 Jan 19]. Available from: https://pubmed.ncbi.nlm.nih.gov/22564397/22564397 10.1016/j.eururo.2012.04.020 PMC3815632

[27] Bandini M, Briganti A, Plimack ER, et al. Modeling 1-year relapse-free survival after neoadjuvant chemotherapy and radical cystectomy in patients with clinical T2–4N0M0 urothelial bladder carcinoma: endpoints for Phase 2 trials. Eur Urol Oncol. 2019;2:248–256. [cited 2021 Jan 19]. Available from: https://pubmed.ncbi.nlm.nih.gov/31200838/31200838 10.1016/j.euo.2018.08.009 PMC7552889

[28] Brimo F, Downes MR, Jamaspishvili T, et al. Prognostic pathological factors in radical cystectomy after neoadjuvant chemotherapy. Histopathology. 2018;73:732–740. [cited 2021 Jan 19]. Available from: https://pubmed.ncbi.nlm.nih.gov/29776013/29776013 10.1111/his.13654

[29] Bandini M, Pederzoli F, Madison R, et al. Unfavorable cancer-specific survival after neoadjuvant chemotherapy and radical cystectomy in patients with bladder cancer and squamous cell variant: a multi-institutional study. Clin Genitourin Cancer. 2020;18:e543–e556. [cited 2021 Jan 19]. Available from: https://pubmed.ncbi.nlm.nih.gov/32144050/32144050 10.1016/j.clgc.2020.01.007 PMC8491463

[30] Lin J, Whalen M, Holder D, et al. Neoadjuvant chemotherapy in the treatment of muscle invasive bladder cancer with mixed histology. Can J Urol. 2013;20:6690–6695. 23587508

[31] Siefker-Radtke AO, Dinney CP, Shen Y, et al. A phase 2 clinical trial of sequential neoadjuvant chemotherapy with ifosfamide, doxorubicin, and gemcitabine followed by cisplatin, gemcitabine, and ifosfamide in locally advanced urothelial cancer: final results. Cancer. 2013;119:540–547. [cited 2021 Jan 19]. Available from: https://pubmed.ncbi.nlm.nih.gov/22914978/22914978 10.1002/cncr.27751 PMC3828072

[32] Vetterlein MW, Wankowicz SAM, Seisen T, et al. Neoadjuvant chemotherapy prior to radical cystectomy for muscle-invasive bladder cancer with variant histology. Cancer. 2017;123:4346–4355. [cited 2021 Jan 19]. Available from: https://pubmed.ncbi.nlm.nih.gov/28743155/28743155 10.1002/cncr.30907

[33] Necchi A, Raggi D, Gallina A, et al. Updated results of PURE-01 with preliminary activity of neoadjuvant pembrolizumab in patients with muscle-invasive bladder carcinoma with variant histologies. Eur Urol. 2020;77:439–446. [cited 2021 Jan 19]. Available from: https://pubmed.ncbi.nlm.nih.gov/31708296/31708296 10.1016/j.eururo.2019.10.026

[34] Blick C, Hall P, Pwint T, et al. Accelerated methotrexate, vinblastine, doxorubicin, and cisplatin (AMVAC) as neoadjuvant chemotherapy for patients with muscle-invasive transitional cell carcinoma of the bladder. Cancer. 2012;118:3920–3927. [cited 2021 Jan 19]. Available from: https://pubmed.ncbi.nlm.nih.gov/22614698/22614698 10.1002/cncr.26675

[35] Choueiri TK, Jacobus S, Bellmunt J, et al. Neoadjuvant dose-dense methotrexate, vinblastine, doxorubicin, and cisplatin with pegfilgrastim support in muscle-invasive urothelial cancer: pathologic, radiologic, and biomarker correlates. J Clin Oncol. 2014;32:1889–1894. [cited 2021 Jan 19]. Available from: https://pubmed.ncbi.nlm.nih.gov/24821883/24821883 10.1200/JCO.2013.52.4785 PMC7057274

[36] Abufaraj M, Foerster B, Schernhammer E, et al. Micropapillary urothelial carcinoma of the bladder: a systematic review and meta-analysis of disease characteristics and treatment outcomes. Eur Urol. 2019:649–658. [cited 2021 Jan 20]. Available from: https://pubmed.ncbi.nlm.nih.gov/30553613/30553613 10.1016/j.eururo.2018.11.052

[37] Moschini M, Shariat SF, Lucianò R, et al. Pure but not mixed histologic variants are associated with poor survival at radical cystectomy in bladder cancer patients. Clin Genitourin Cancer. 2017;15:e603–e607. Available from: https://pubmed.ncbi.nlm.nih.gov/28040422/28040422 10.1016/j.clgc.2016.12.006

[38] Dayyani F, Czerniak BA, Sircar K, et al. Plasmacytoid urothelial carcinoma, a chemosensitive cancer with poor prognosis, and peritoneal carcinomatosis. J Urol. 2013;189:1656–1661. Available from: https://pubmed.ncbi.nlm.nih.gov/23159581/23159581 10.1016/j.juro.2012.11.084 PMC4243847

[39] Berg S, D’Andrea D, Vetterlein MW, et al. Impact of adjuvant chemotherapy in patients with adverse features and variant histology at radical cystectomy for muscle-invasive carcinoma of the bladder: does histologic subtype matter? Cancer. 2019;125:1449–1458. [cited 2021 Jan 20]. Available from: https://pubmed.ncbi.nlm.nih.gov/30620387/30620387 10.1002/cncr.31952

[40] Krasnow RE, Drumm M, Roberts HJ, et al. Clinical outcomes of patients with histologic variants of urothelial cancer treated with trimodality bladder-sparing therapy. Eur Urol. 2017;72:54–60. [cited 2021 Jan 20]. Available from: https://pubmed.ncbi.nlm.nih.gov/28040351/28040351 10.1016/j.eururo.2016.12.002

[41] Choi W, Porten S, Kim S, et al. Identification of distinct basal and luminal subtypes of muscle-invasive bladder cancer with different sensitivities to frontline chemotherapy. Cancer Cell. 2014;25:152–165. [cited 2021 Jan 20]. Available from: https://pubmed.ncbi.nlm.nih.gov/24525232/24525232 10.1016/j.ccr.2014.01.009 PMC4011497

[42] Tse J, Ghandour R, Singla N, et al. Molecular predictors of complete response following neoadjuvant chemotherapy in urothelial carcinoma of the bladder and upper tracts. Int J Mol Sci. 2019. 10.3390/ijms20040793 PMC6413224 30781730

[43] Jain RK, Sonpavde G. Neoadjuvant therapy for muscle-invasive bladder cancer. Expert Rev Anticancer Ther. 2020:603–614. [cited 2021 Jan 20]. Available from: https://pubmed.ncbi.nlm.nih.gov/32546025/. 32546025 10.1080/14737140.2020.1784011

